# Aminoglycoside Therapy for Tuberculosis: Evidence for Ototoxicity among Tuberculosis Patients in Ghana

**DOI:** 10.3390/diseases10010010

**Published:** 2022-02-01

**Authors:** Enid Owusu, Benjamin T. Amartey, Emmanuel Afutu, Neal Boafo

**Affiliations:** 1 Department of Medical Laboratory Sciences, School of Biomedical and Allied Health Sciences, University of Ghana, Accra 233, Ghana; 2 Department of Audiology, Speech and Language Therapy, School of Biomedical and Allied Health Sciences, University of Ghana, Accra 233, Ghana; btamartey@gmail.com (B.T.A.); nealb7777@gmail.com (N.B.); 3 Department of Medical Microbiology, School of Biomedical and Allied Health Sciences, University of Ghana, Accra 233, Ghana

**Keywords:** tuberculosis, aminoglycosides, anti-TB, hearing impairment

## Abstract

Background: Hearing impairment (HI) is a major problem in Ghana; however, the few attempts at shedding light on its causes appear to overlook the adverse effects of some medications—a gap that this study sought to fill. Aminoglycoside therapy for tuberculosis (TB) treatment is one of these medications. Aim: The aim of this study was to establish the potential of aminoglycoside as a cause of hearing impairment among patients on anti-TB treatment. Method: This was a case–control study, involving patients receiving treatment for TB with aminoglycoside at the chest clinic of the Tema General Hospital and a control group of age- and gender-matched healthy volunteers. A structured questionnaire was administered to obtain the demographic data and case history of the participants. The hearing sensitivity of the participants was assessed using conventional pure tone audiometry and transient evoked otoacoustic emission tests. Results: A hearing loss prevalence of 20% (12/60) was found among patients receiving treatment for TB, with the intensity of impairment ranging from mild to severe. Hearing thresholds of patients receiving anti-TB medications were significantly elevated (*p* < 0.05) in comparison to the thresholds of the control group, especially at the high frequencies. Conclusion: This study shows that aminoglycoside therapy for tuberculosis may contribute to hearing impairment among tuberculosis patients in Ghana. Audiological management of these patients should therefore be an essential part of their therapeutic treatment plan.

## 1. Introduction

Hearing impairment (HI) is one of the notable disabling conditions of significant global health concern that can have a detrimental effect on a country’s socio-economic development, if not addressed properly. According to the World Health Organization (WHO) [1], unless action is taken, by 2030 there will be nearly 630 million people with disabling hearing loss, and by 2050, the number could rise to over 900 million.

This is evident in Ghana, where a report from Komfo Anokye Teaching hospital (KATH) showed that there is an overall increase in the number of patients with hearing loss who visited the hospital between 1999 and 2004 [2]. In 2006, Awuah and colleagues, after screening patients suffering from different forms of ENT disease who visited the KATH, reported an HI prevalence of 91.3% [3]. In Accra, a similar study also showed 66.3% of patients who visited the Korle-Bu Teaching Hospital (KBTH) in the year 2013 to be diagnosed with HI [4].

Even though HI is unquestionably a public health problem, there appears to be little effort to investigate it in Ghana. There are currently no comprehensive studies on the genetic and environmental etiologies, classification, and burden of HI on Ghana, with only little evidence existing on HI in patients receiving ototoxic treatments in Ghana. For example, in a community-based survey among children in peri-urban Kumasi, Larsen-Reindorf et al. [5] concluded that pediatric hearing loss is prevalent in Ghana and should be treated as a public health problem warranting further evaluation and epidemiology characterization. In another study, after investigating the characteristics of hearing impairment among patients in Ghana, Amedofu et al. [2] showed that factors such as noise, meningococcal meningitis, presbycusis, mumps, and Meniere’s disease were the major causes of sensorineural HI. They further stated that the causes of conductive hearing loss among the patients studied were wax, foreign bodies, otitis media, Eustachian tube malfunction, trauma, and otitis externa. According to them [2], mixed hearing loss was due to mainly a long-standing otitis media and trauma due to accidents or slaps on the ear [2].

Recent studies in the field of HI in Ghana, which tried to identify malaria and sickle cell disease as possible causes of HI in children, suggested that sickle cell disease is not a likely cause of HI or delay in speech in children [6]. Severe malaria, on the other hand, was suggested to influence the function of the inner ear in children; however, the loss of hearing caused by malaria is reversed after treatment. Noise exposure is another major cause of nongenetic HI in Ghana. The noise generated at gold mines, quarries, mills, and other noisy industrial areas is mostly far above the normal levels, predisposing workers to the risk of acquiring HI [7]. There also was empirical evidence that excessive noise generated by the quarries caused 56% of steel/metal workers and communities saturated with a lot of religious noise in Ghana to be at higher risk of developing HI [8]. Meanwhile, there seems to be no attention given to the side effects of some drugs with regard to HI—for example, aminoglycoside therapy for tuberculosis treatment—a gap this study sought to fill.

The World Health Organization (WHO) reports that, globally, an estimated 10.4 million people acquired tuberculosis (TB) in 2016 [9], with Ghana among the TB endemic regions in sub-Saharan Africa [10]. Co-morbidity with HIV–AIDS as well as the emergence of resistant TB strains (multidrug-resistant TB (MDR-TB) and extensively drug-resistant TB (XDR-TB)) have fueled the disease burden, making it a major threat to disease control and an important public health concern.

The treatment of TB and MDR-TB is largely dependent on the simultaneous administration of multiple antibiotics, including aminoglycosides such as streptomycin and rifampicin. For decades aminoglycosides have been added to other antibiotics for TB therapy because they have been found to be effective. Like most drugs, however, aminoglycosides have been on record to have ototoxic effect on users, which leads to hearing impairment [11,12,13]. Ototoxicity is the result of the exposure of the inner ear to drugs and therapeutic agents, leading to hearing impairment [14].

This side effect has been reported to influence non-compliance and non-adherence to TB therapy [12,15]. This is an important public health problem because it can adversely impair quality of life. Some socioeconomic consequences of hearing impairment include stigma, isolation, frustration, feelings of loneliness, low educational attainment, low income, underemployment, and unemployment [9].

In many jurisdictions, the effect of aminoglycosides on hearing has been well documented [11,12,13], and outcomes from these studies have helped with the management of TB. However, very little work has been done to establish the possible link between aminoglycosides and hearing loss among patients in Ghana, which is a resource limited country. Currently, in Ghana, hearing assessment is not a vital part of the management of patients on an anti-TB treatment regimen as there is a paucity of research data to support policies on the audiometric assessment of these patients.

It is critical, therefore, that this study was conducted, to help identify and document the prevalence of aminoglycoside-associated hearing impairment in study patients who had previously demonstrated no indications of hearing loss before the treatment. It was therefore hypothesized that there will be a significant difference between the hearing thresholds of patients receiving treatment and those not on therapy in the high frequencies. Secondly, it was hypothesized that there will be a significant association between TB therapy and elevated hearing thresholds.

## 2. Materials and Methods

### 2.1. Study Design, Population, and Data Gathering Tools

This was a case–control study (with a non-equivalent control group) design involving 60 consenting patients receiving aminoglycoside treatment therapy (streptomycin and rifampicin) for TB at the chest clinic of the Tema General Hospital (case) and a control group of 60 age- and gender-matched uninfected volunteers.

Patients on TB treatment for less than a month, those presenting with ear infections, and those with history of ototoxic medications within the last 3 months were excluded from the study. The study was conducted at the Chest and Otolaryngology (Ear, Nose, and Throat) clinics of the Tema General Hospital. Only participants who consented to partake after extensive explanation concerning the objectives and significance of the study were enrolled. A structured questionnaire was administered to gather key patient information such as the demographics and clinical histories, including case history information and HIV–AIDS comorbidity.

### 2.2. Assessment Methods

#### 2.2.1. Otoscopic Examination

Participants had their ears preliminarily assessed for abnormalities such as for signs and presence of foreign bodies, tympanic membrane perforation, impacted wax, obstructions in the external ear canal, and collapsed ear canals.

#### 2.2.2. Puretone Audiometry (PTA)

Using a calibrated Otovation Amplitude T3 Diagnostic PC-based audiometer with EAR 5A inserts and Radiocar B-71 bone conductor, the conventional (250–8000 Hz) Puretone Audiometry (PTA) was conducted on all participants who underwent and passed otoscopic examination. Functional inspection, daily biological calibration, and performance checks were done on the audiometer. With the appropriate insert ear tip size, study participants were fitted with the insert earphone. Testing began in the better hearing ear (according to the participants’ account) at 30 dBHL at 1000 Hz, using an ascending approach. Next, 2000 Hz, 3000 Hz, 4000 Hz, 6000 Hz, 8000 Hz, 500 Hz, and 250 Hz were tested in that order. To validate the threshold, testing was redone at 1000 Hz. The contralateral ear was tested using the same order except that 1000 Hz was not retested to validate the thresholds.

The tests were conducted using standard audiometric procedures [16]. With bone conduction testing, the bone vibrator was placed over the mastoid prominence of the worse ear. It was placed exactly behind the pinna and caution was exercised to prevent the instrument touching the pinna or hair. The vibrator was held firmly by a headband. Bone conduction testing was also done using standard audiometric procedures [16]. Masking was performed using standard audiometric procedures whenever any of these three (3) indications were realized: when the difference between the right and left unmasked AC thresholds was 55 dB or more; when there is an air–bone gap of 10 dB and above; and when the first condition has not been applied, but the difference between the BC threshold of one ear and the unmasked AC threshold of the other ear is 55 dB or more [16].

The degrees of hearing loss across subjects were determined using the pure-tone average of the air conduction thresholds of subjects at 500 Hz, 1000 Hz, and 2000 Hz. They were classified using the commonly used modification of the combination of the Goodman (1965) and Clark (1981) classifications [17]. Normal hearing was defined as (air-conducted) pure tone thresholds of 25 dBHL or better at octave frequencies from 250 Hz to 4000 Hz in both ears and degrees of hearing loss were determined using (air-conducted) pure-tone thresholds at octave frequencies from 500 Hz to 4000 Hz in each ear. Hearing loss was present if the PTA threshold was >25 dBHL. For classification of the degree of hearing loss, 26–40 dBHL was described as “Mild”, 41–55 dBHL was described as “Moderate”, 56–70 dBHL was described as “Moderately-severe”, 71–90 dBHL was described as “Severe”, and 91 dBHL upwards was considered “Profound” degree of hearing loss [17].

#### 2.2.3. Otoacoustic Emissions

The status of the hair cells in the cochlea of participants was assessed using the Echo-screen TS SET transient-evoked otoacoustic emissions (TEOAE) test. In brief, a probe with a miniature loudspeaker and a microphone was inserted into the ears of the participant. A click or tone burst stimulus was applied to the participant’s ears via the loudspeaker in the probe. The cochlea then responds to the acoustic stimulus by generating sound waves that are then picked up, measured, recorded, and analyzed by the microphone in the probe. The outcomes were categorized into “pass” or “refer” depending on the integrity of the cochlea, based on the criteria described in Table 1. For a participant to pass, the results should satisfy the following criteria in two ears: (1) the overall reproducibility should be >50%; and (2) the reproducibility and signal-to-noise ratio (SNR) should be as specified in the table for each frequency band. The results were automatically reported as “pass” or “refer” on the TEOAE device. A “pass” indicates that the reproducibility and SNR criteria were met at 1.5 kHz–4 kHz for at least 2 frequency bands. A “refer” indicates that both criteria were not met at 1.5 kHz and 4 kHz for at least 2 frequency bands.

### 2.3. Statistical Analysis

Data from study were preliminarily described as percentages and frequencies to determine the prevalence of the condition. A Chi-square test was performed to reveal any association between the exposure to TB medications and the auditory thresholds between the two groups. Statistical significance was defined as *p* < 0.05. All statistical analyses were conducted using Statistical Package for Social Sciences (SPSS), version 20.

### 2.4. Safety and Ethical Considerations

This study enrolled TB-infected individuals and TB is known to be highly infectious; thus, this study adhered to stringent safety measures, as recommended by Kemp and Bankaitis [18], to curb cross infection. Some of the measures included regular handwashing, use of personal protective wear (PPE), appropriate disposal methods, and cleaning and sterilization of the OAE probe and otoscopic specula and other items. Ethical clearance was granted by the Ethics and Protocol Review Committee (EPRC) of the School of Biomedical and Allied Health Sciences (SBAHS) with approval permit number SBAHS-ASLT/10241594/SA/2017-2018. Permission and authorization were granted for the data collected by the Authorities of the Tema General Hospital.

## 3. Results

### 3.1. Demographic Characteristics of the Study Participants

A total of 120 study participants was enrolled in the study. This was made up of the treatment group (n = 60) and control group (n = 60), aged between 18 and 50 years with mean ages of 37.97 years and 34.17, respectively. The minimum age of the participants enrolled in the study was 18 years whilst with the maximum was 50 years. Participants within the age range of 40–50 years were the most prevalent in the treatment group (n = 29, 48%), whilst the least prevalent (n = 13, 23%) was the age bracket of 18–29 years (Table 2). For the control group, the most prevalent age range was 18–29 years (n = 22, 36%). Participants who presented with pulmonary TB in the treatment group were more prevalent (n = 49, 81.7%) compared to those who presented with extra-pulmonary TB (n = 11, 18.3%). The majority of the participants in the treatment group were HIV negative (n = 54, 90%) (Table 2).

### 3.2. Clinical Characteristics of Treatment Group

In the treatment group, new cases of TB were the most prevalent (n = 46, 76.7%) compared to retreatment cases (n = 14, 23.3%) (Table 3). The last phase of TB treatment was more prevalent (n = 40, 66.7%) than the first phase of treatment (n = 20, 33.3%). Data on the duration of treatment revealed that 1–2 months and 3–4 months were more prevalent (n = 18, 30% each), whereas 9–12 months (n = 1, 1.7%) was the least prevalent (Table 3).

### 3.3. Distribution of Audiometric Data

#### 3.3.1. Distribution of Maximum and Minimum Pure Tone AC and BC Thresholds

Table 4 and Table 5 present a breakdown of the maximum and minimum pure tone BC and AC thresholds compared to the overall mean and standard deviation at the test frequencies in both the treatment and control groups. The highest means and standard deviation of the AC and BC thresholds in the treatment group were recorded (AC: right ear = 18.71 ± 12.3, left ear = 19.58 ± 11.66; and BC: right ear = 37.50 ± 8.39, left ear = 39.17 ± 9.25) at 8000 Hz and 4000 Hz, respectively. The means and standard deviation for the lowest AC thresholds were recorded at 250 Hz (right ear = 15.67 ± 6.28, left ear = 16.92 ± 5.62). The highest means and standard deviations for the AC and BC thresholds in the treatment group were recorded at high frequencies (2–8 KHz).

#### 3.3.2. Degrees of Hearing Loss in the Treatment and Control Groups

**Figure 1:**
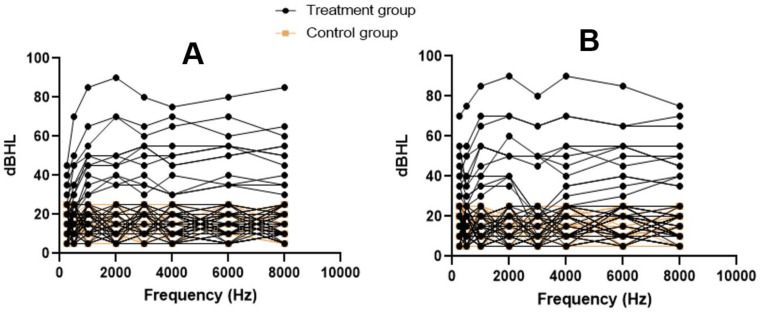
Spaghetti plots of the individual audiograms of the participants: (**A**) right ear; (**B**) left ear.

Individual audiograms for participants are presented as spaghetti plots for the right and left ears in Figure 1. Among the participants in the treatment group, 48 (80%) presented with normal hearing thresholds whilst 12 (20%) presented with varying degrees of SNHL. They consisted of 4 (6.7%) participants with mild SNHL, 5 with moderate (8.3%), 2 with moderately severe (3.3%), and 1 with severe (1.7%). None of the participants presented with profound SNHL and a normal hearing threshold was recorded in 48 (80%) of the participants in the treatment group. The most prevalent degree of loss was moderate SNHL (n = 5, 8.3%), with the least prevalent being severe SNHL. The SNHL recorded were all bilateral and symmetrical in all the participants who presented with hearing loss. All the participants recruited into the control group presented with normal hearing thresholds (n = 60, 100%).

#### 3.3.3. Distribution of OAE Results

**Figure 2:**
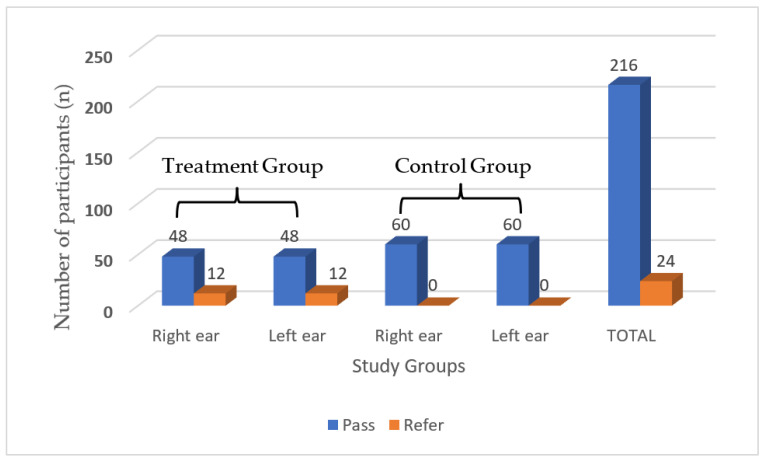
Distribution of the OAE results for both ears in the treatment and control groups.

The OAE results for the participants in the treatment and control groups are displayed in Figure 2. Findings indicated that 48 (80%) participants in the treatment group presented with passes in both ears whereas 12 (20%) participants were referred in both ears (Figure 2). All participants in the control group, however, recorded passes in both ears. Overall, out of the 240 ears assessed, 216 (90%) recorded passes in both groups whilst 24 (10%) ears recorded referrals (Figure 2).

### 3.4. Differences in the Treatment and Control Groups

Differences in the means of the treatment and control groups were tested using an independent t-test. Statistically, the treatment group differed significantly (*p* < 0.05) from the control group with respect to the mean hearing thresholds at the high frequencies (2000, 3000, and 4000 Hz) in the two ears. The mean hearing threshold of the treatment group was greater than that of the control group at the 0.05 level of significance. The breakdown of the independent t-test on the mean differences between the treatment and control groups is presented in Table 6.

### 3.5. Association between the Audiometry Findings and Exposure to TB Medications

For the association between the audiometry findings and exposure to TB medications, a Chi-square test was performed. The results revealed a statistically significant association between abnormal audiometry findings and exposure to TB medications (χ^2^ = 13.33, *p* < 0.05), as presented in Table 7.

## 4. Discussion

Despite hearing impairment (HI) clearly being a public health issue, there seems to be just some modest effort to study its causes in Ghana. Contrary to the situation in Ghana, in many jurisdictions, the effect of aminoglycosides on hearing has been well documented [11,12,13], and results from these studies have helped with the management of TB. Currently, a hearing assessment is not a vital part of the management of patients on an anti-TB treatment regimen in Ghana, as there is not enough research data to support policies on the audiometric assessment of these patients. This study therefore sought to fill that knowledge gap by helping to document the prevalence of aminoglycoside treatment-associated hearing impairment and the ototoxicity among TB patients who had previously shown no signs of hearing loss before the aminoglycoside treatment. 

The present study showed a hearing impairment prevalence of 20% among patients on aminoglycosides treatment. The proportion of patients who had their hearing affected in this study is consistent with the findings reported in other studies conducted all over the world. These included findings such as 18.75% reported by Duggal and Sarkar [19]; 36.83% by Javaid et al. [20] in Asia; 18% and 28% reported by De Jager and Van Altena [21] and Sturdy et al. [22], respectively, in Europe; 23.7% by Vasconcelos et al. [23] in South America; 37% by Peloquin et al. [24] in North America; 47% by Ramma and Ibekwe [25] and 58% by Sagwa et al. [26], both in southern Africa; and 61% and 22.9% reported by Ibekwe and Nwosu, [27] and Sogebi et al. [28], respectively, in West Africa. The wide variation in the hearing loss prevalence could be ascribed to the difference in geographical locations and in the audiology test used by the various studies. Whereas conventional pure tone audiometry was used in this study, most of the other studies employed high frequency pure tone audiometry, which can assess hearing above 8000 Hz.

The occurrence of hearing impairment among patients on aminoglycosides treatment is backed by studies that have described evidence of the presence of aminoglycosides in the inner ear [29,30,31]. Aminoglycosides have been said to enter the inner ear fluids via the bloodstream following parenteral administration and their presence is felt in the inner ear within a couple of minutes and may plateau within 30 min to 3 h following systemic administration [29,30]. Huy et al. [31] identified the delayed presence of aminoglycosides in inner ear tissues long after the bloodstream has been rid of the drug. Despite having a shelf-life of 3–5 h in serum, aminoglycosides, however, have been described to stay longer in the inner ear fluids, even months after treatment has ended. This explains the occurrence of post-treatment delayed hair cell death [29,31]. In the cochlea, aminoglycosides can be found in the hair cells and in some supporting cells. However, although these drugs reach the inner ear shortly after administration, it takes several days before hair cell destruction starts [32,33]. Once aminoglycosides enter the inner ear, they bind with ions, which results in the formation of free radicals known as reactive oxygen species (ROS). This ROS activates and triggers active signal pathways that result in cell death from apoptosis. This apoptosis permanently destroys the outer hair cells, leading to irreversible hearing loss. It has been said that the initial effects of the aminoglycoside-induced hearing loss occur in the outer hair cells at the basal portion of the cochlea where high frequencies are processed and then progresses to the apical portion of the cochlea that processes low frequencies [29,30,33,34,35,36,37].

The high prevalence of hearing loss reported among TB patients undergoing treatment in this study can be attributed to the fact that 50% of the patients presented with hearing loss were also TB–HIV co-infected. This finding is consistent with findings from a study by Harris et al. [38], who established that the probability of developing hearing loss was higher (about 4 times) in TB–HIV co-infected patients compared to HIV-negative patients. It may be that patients treated with both aminoglycoside and ART exhibit synergistic effects. As a result, the cases of TB–HIV co-infection in this study may possibly disturb the picture that the hearing impairment observed among the patients is only due to tuberculosis treatment.

A significant proportion of the patients who initiated TB treatment presented with poor hearing thresholds in this study. This finding is consistent with works by Törün et al. [11], Gülbay et al. [12], and Yang et al. [13], who unanimously established that ototoxic hearing loss is a common and frequently observed side effect experienced by patients undergoing TB treatment. Another study that supported this finding was conducted by Mwansasu et al. [39], who investigated the degree of hearing loss among TB patients on therapy. They concluded that a substantial proportion of patients with TB initiated on an anti-TB regimen ended up with significant hearing loss. 

The poor thresholds reported in this study were all bilateral and evident in the high frequencies, which is consistent with findings by Sagwa et al. [26], Nizamuddin et al. [40], Sharma et al. [41], and Tiwari et al. [42]. These studies concluded that ototoxic hearing loss is bilateral and is evident in the high frequencies. In addition, the majority of the patients on retreatment in this study presented with hearing loss and this is consistent with findings by Modongo et al. [43], who postulated that a longer treatment duration was associated with the development of hearing loss.

The poorest hearing thresholds in this study were recorded by patients on an anti-TB regimen since the mean and standard deviations of the AC thresholds of the treatment group were higher than in the controls. This result is consistent with findings from a study conducted by Brits et al. [44], who compared the hearing status of gold miners with and without TB.

Higher hearing threshold differences were recorded in the higher frequencies than in the lower frequencies between the treatment and control groups. Aminoglycoside-induced hearing loss has been observed with a distinct pattern, which shows the high frequencies (4000–8000 Hz) being affected first with the lower frequencies affected later [45]. In this study, the higher hearing threshold differences recorded in the higher frequencies than in the lower frequencies between the treatment and control group is congruous with findings by Huth et al. [30], Karasawa and Steyger [36], Tabuchi et al. [37], and Sha et al. [46], who concluded that the base of the cochlea where high frequencies are transduced are more vulnerable than the apical end where low frequencies are transduced. This explains why high frequency losses precede low frequency losses in drug-induced hearing losses. It also explains why hearing thresholds at higher frequencies in the present study were remarkably different between the two groups.

It was hypothesized that there will be a significant difference between the hearing thresholds of patients receiving treatment and those not on therapy in the high frequencies. This hypothesis was supported by the data collected. Differences with respect to hearing thresholds in the group means were noted in all frequency categories. However, the differences that were statistically significant were found between the treatment and control groups in the high frequencies. This hypothesis is consistent with the findings from a study conducted by Brits et al. [44], who reported that a highly significant difference exists between the hearing thresholds of patients undergoing treatment for TB and those not infected with TB.

The study also revealed a significant elevation in the hearing thresholds of the single and multiple treatment groups compared to those of the controls. However, there were no significant differences in the mean hearing threshold of TB patients on treatment and uninfected persons in the low frequency categories. In addition, Brits et al. [44] also reported that differences in thresholds were more pronounced in the high frequencies compared to the low frequencies, and this agrees with the findings from the present study. This hypothesis is also supported by a study conducted by Vasconcelos et al. [23], in which irreversible hearing losses ranging from mild to severe were reported among patients initiated on TB medications. The findings also revealed that auditory thresholds were worse at the high frequencies.

The second hypothesis stated that there will be a significant association between aminoglycosides therapy and elevated hearing thresholds. This was also supported by the data collected. A test of association between hearing status and the two group revealed a statistically significant association (*p* < 0.05) between the treatment group and OAE referral. All the patients referred in the OAE and who also demonstrated hearing loss of varying degrees were found to be in the treatment group and also undergoing therapy. Another test of association conducted to explore the association between the audiometry findings and exposure to TB medications also reported a statistically significant association between the abnormal audiometry findings and exposure to TB medications. 

These results are consistent with findings from studies conducted by Duggal and Sarkar [19], Sagwa et al. [26], Ibekwe and Nwosu [27], and Sogebi et al. [28], who compared the baseline hearing thresholds with post-treatment hearing thresholds of patients initiated on an anti-TB regimen. Their findings revealed that most of the patients presented with elevated thresholds at the end of the therapy. Based on this, they unanimously concluded that TB therapy is associated with reduced hearing thresholds. The hypothesis was also supported by a study conducted by Mwansasu et al. [39], who established that a significant proportion of patients enrolled on TB therapy end up demonstrating poorer hearing thresholds. Sagwa et al. [26] also concluded that reduced hearing thresholds in patients with TB are an outcome of TB treatment. 

## 5. Conclusions

This study has shown that aminoglycoside therapy for tuberculosis may play a role in HI in Ghanaian patients. The study revealed elevated hearing thresholds among the patient’s receiving treatment for TB as compared to the controls. The patients presented with various degrees of hearing losses and this finding is consistent with findings from studies that have been conducted in other parts of the world. This study based on outcomes obtained concluded that patients on aminoglycoside therapy are more likely to end up with significant hearing impairment due to the side effects of the treatment regimen. Audiological management of these patients should therefore be an essential part of their therapeutic treatment plan, to minimize the socio-economic effect they suffer just because of their willingness to get treated.

## 6. Limitations

Although some limitations can be identified in the study, this did not significantly affect the outcome and interpretations. These limitations included the use of a small sample size, the inability to perform tympanometry, extended high frequency audiometry and diagnostics DPOAE, and the inability to conduct pretreatment measurements and ascertain treatment compliance. Furthermore, no secondary analysis was conducted to account for patients treated with antiretroviral drugs for HIV, even though there is some preclinical data to suggest that these medications may also lead to ototoxicity, possibly disturbing the picture of only tuberculosis treatment being the reason for hearing damage. Additionally, the definition of PTA used in this study can be considered old, even though that is what is commonly used in Ghana and as such was applied in the current study in order to give a better representation of the situation in the country. Nonetheless, the outcomes from this study underscore the need for the auditory monitoring of patients initiated on anti-TB therapy because of ototoxicity concerns.

## Figures and Tables

**Table 1:** Pass criteria for transient evoked otoacoustic emissions (TEOAE) screening.

Frequency Band (kHz)	Signal to Noise Ratio (dB)	Reproducibility (%)
1.5	≥+3	≥50
2.0	≥+6	≥70
3.0	≥+6	≥70
4.0	≥+6	≥70

**Table 2:** Gender, age, TB, and HIV status of the participants.

Demographic Variable		Treatment Group		Control Group		Total	
		n	%	n	%	n	%
Gender	Male	27	45	27	45	54	45
	Female	33	55	33	55	66	55
Age (years)	18–29	13	23	22	36	35	29
	30–39	18	30	19	32	37	31
	40–50	29	48	19	32	48	40
TB Type	ETB	11	18.3			11	18.3
	PTB	49	81.7			49	81.7
	Total	60	100			60	100
HIV Status	Positive	6	10			6	10
	Negative	54	90			54	90

n: frequency; %: percentage; TB: tuberculosis; ETB: extra-pulmonary TB; PTB: pulmonary TB.

**Table 3:** Clinical characteristics of the treatment group.

Variable		Treatment Group	
		Number (n)	Percentage (%)
Cases	New	46	76.7
	Retreatment	14	23
	Total	60	100
Phases	First phase	20	33.3
	Last phase	40	66.7
Treatment duration	1–2 months	18	30
	3–4 months	18	30
	5–6 months	11	18.3
	7–8 months	12	20
	9–12 months	1	1.7

**Table 4:** Distribution of the maximum and minimum pure tone bone-conduction thresholds compared to the overall mean and standard deviation at test frequencies in the treatment group.

Bone Conduction Thresholds dBHL				
Treatment Group				
Frequency (Hz)	Ears	Maximum	Minimum	Mean ± SD
500	Right Left	30 35	15 15	22.50 ± 5.87 22.50 ± 6.91
1000	Right Left	40 30	15 15	26.67 ± 7.79 25.83 ± 6.34
2000	Right Left	40 40	10 25	29.17 ± 7.64 32.50 ± 4.52
4000	Right Left	55 55	30 30	37.50 ± 8.39 39.17 ± 9.25

**Table 5:** Distribution of the maximum and minimum pure tone air-conduction thresholds compared to the overall mean and standard deviation at test frequencies in both the treatment and control groups.

Air Conduction Thresholds dBHL							
		Treatment Group			Control Group		
Freq (Hz)	Ears	Maximum	Minimum	Mean ± SD	Maximum	Minimum	Mean ± SD
250	Right Left	30 30	5 5	15.67 ± 6.28 16.92 ± 5.62	25 25	5 5	15.33 ± 5.44 17.00 ± 4.71
500	Right Left	30 30	5 5	15.63 ± 6.30 17.33 ± 5.46	25 25	5 10	15.33 ± 5.81 16.67 ± 4.19
1000	Right Left	40 30	5 5	16.79 ± 7.24 16.75 ± 6.07	25 25	5 5	16.17 ± 5.92 16.58 ± 4.74
2000	Right Left	40 45	5 5	15.83 ± 7.54 16.79 ± 7.12	25 25	5 5	15.00 ± 6.18 15.33 ± 4.86
3000	Right Left	45 45	0 5	17.21 ± 8.09 17.46 ± 8.10	25 25	5 5	15.75 ± 6.16 16.67 ± 4.84
4000	Right Left	60 55	0 0	17.25 ± 9.99 18.25 ± 9.61	25 25	0 5	15.58 ± 5.83 17.58 ± 4.56
6000	Right Left	80 75	0 0	17.92 ± 11.4 18.88 ± 11.1	25 25	0 5	15.83 ± 6.19 16.92 ± 4.97
8000	Right Left	80 80	5 0	18.71 ± 12.3 19.58 ± 11.7	25 25	5 0	15.75 ± 6.02 18.33 ± 5.94

dB = decibels; HL = hearing loss; Sd = standard deviation; Hz = Hertz.

**Table 6:** Independent t-test on the mean differences between the treatment and control groups’ hearing thresholds.

Hearing THRESHOLD Parameter (Hz)	Ear	Treatment Group Mean ± SD	Control Group Mean ± SD	*p*-Value
2000, 3000, 4000	Right Left	18.68 ± 9.03 18.93 ± 9.60	15.70 ± 4.093 15.90 ± 3.740	0.021 0.024
500, 1000, 2000	Right Left	16.95 ± 6.98 17.70 ± 6.53	15.50 ± 3.96 16.17 ± 3.45	0.164 0.111

α = 0.05; dB = decibels; Hz = Hertz; Sd = standard deviation.

**Table 7:** Chi-square test for association between audiometry findings and exposure to TB medications.

Variable	Normal Audiometry Findings		Abnormal Audiometry Findings		χ^2^	df	*p*-Value
	n	%	N	%			
On medication	48	44.4	12	100	13.33	1	0.000
No medication	60	55.6					

α = 0.05.

## Data Availability

All data supporting the results have been included in the paper.
